# The importance of cultural tailoring of communicators and media outlets in an influenza vaccination awareness campaign: a digital randomized trial

**DOI:** 10.1038/s41598-023-27910-y

**Published:** 2023-02-16

**Authors:** G. L. Habib, H. Yousuf, L. Bredius, N. R. Bindraban, M. M. Winter, E. J. A. Scherder, S. van der Linden, J. Narula, L. Hofstra

**Affiliations:** 1 Department of Cardiology, Amsterdam UMC, Amsterdam, The Netherlands; 2 Department of Clinical Neuropsychology, VU, Amsterdam, The Netherlands; 3 Department of Psychology, School of Biology, University of Cambridge, Cambridge, UK; 4 Mount Sinai Heart, Icahn School of Medicine at Mount Sinai, New York, NY USA

**Keywords:** Human behaviour, Influenza virus, Randomized controlled trials, Preventive medicine

## Abstract

The COVID-19 pandemic has exposed the vulnerability of ethnic minorities again. Health inequity within ethnic minorities has been explained by factors such as higher prevalence of underlying disease, restricted access to care, and lower vaccination rates. In this study, we investigated the effect of cultural tailoring of communicators and media outlets, respectively, on vaccine willingness in an influenza vaccination campaign in the Netherlands. A total of 1226 participants were recruited from two culturally non-tailored media outlets (Dutch newspaper and Facebook), and one media outlet tailored to a large community in the Netherlands with Indian ancestry. The participants from all three media outlets were randomly exposed to a vaccination awareness video delivered by a physician with an Indian or Dutch background, followed by an online survey. Cultural tailoring compared to cultural non-tailoring of communicators showed no difference in improvement of vaccine willingness (13.9% vs. 20.7% increment, respectively, *p* = 0.083). However, the media outlet tailored to the community with Indian ancestry, resulted in a higher improvement of vaccine willingness compared to non-tailored media outlets (46.7% vs. 14.7% increment, respectively, *p* < 0.001, unadjusted OR = 5.096). These results suggest that cultural tailoring of media outlets may be critical to effectively reach out to ethnic minorities to help optimize vaccination rates and improve general health.

## Introduction

The average global burden of influenza is high and varies with the virulence of the Influenza strain. Influenza vaccination is not only an effective measure to prevent respiratory complications^1^, but could be even more effective than statins or antihypertensives for prevention of acute myocardial infarction^2^. Given the importance of this vaccine effectiveness, we were previously invited by the Netherlands Society of Cardiology to establish a call to action to cardiologists to promote influenza vaccination^3^. According to the annual Dutch flu vaccine campaign from 2021, vaccine coverage among people aged 65 years or older comprised 72.0%^4^, which is approximating the indicated numbers by the WHO***,*** who advocates for an Influenza vaccination coverage of 75% among older people^5^.

However, ethnic minorities have consistently shown lower vaccination rates. For instance, according to the Centers for Disease Control and Prevention (CDC), in the 2019–2020 flu season in the US, the white population had the highest vaccination coverage (53%), compared to non-Hispanic Black (41%), Hispanic (38%) and other population groups^6^. The ethnic minorities have also universally demonstrated vaccine hesitancy towards the COVID-19 vaccine, and governments have struggled to better protect these vulnerable groups^7^. Also in the Netherlands people with a migration background, including citizens who migrated from Suriname, were less willing to get vaccinated against COVID-19, more often admitted to the hospital, and showed a 1.5 times higher mortality rate per 100.000 persons^8^. These data are in line with the results of a large survey demonstrating higher vaccine hesitancy in the population with a migration background in the Netherlands^9^. However, with respect to the influenza vaccination, data on vaccine uptake are lacking, since migration background is not registered according to the annual Dutch flu vaccine campaigns in 2019 to 2021^4,10,11^.

Health inequity within ethnic minorities has been explained by factors such as the higher burden of underlying disease, limited access to health care, social deprivation, and health illiteracy^12,13^. It has been reported that COVID-19 health messages from a race or ethnic messenger aligned to the audience helped improve information-seeking behavior, specifically in Black participants^14^, and that a comprehensive coordinated community strategy tailored to the target population, resulted in the highest vaccination rate among American Indians (AI) and Alaskan Natives (AN) in the USA^15^.

However, the question remains as to which specific elements of a comprehensive communication strategy define the success^15^. We have previously demonstrated the success of a data-driven public health campaign on hygiene, and also the effectiveness of a media intervention for debunking of vaccination myths^16–18^. In the current study, we investigated the efficacy of cultural tailoring at the level of health communicators and at the level of media outlets on vaccine acceptance for an influenza vaccination awareness campaign.

## Results

**Figure 1:**
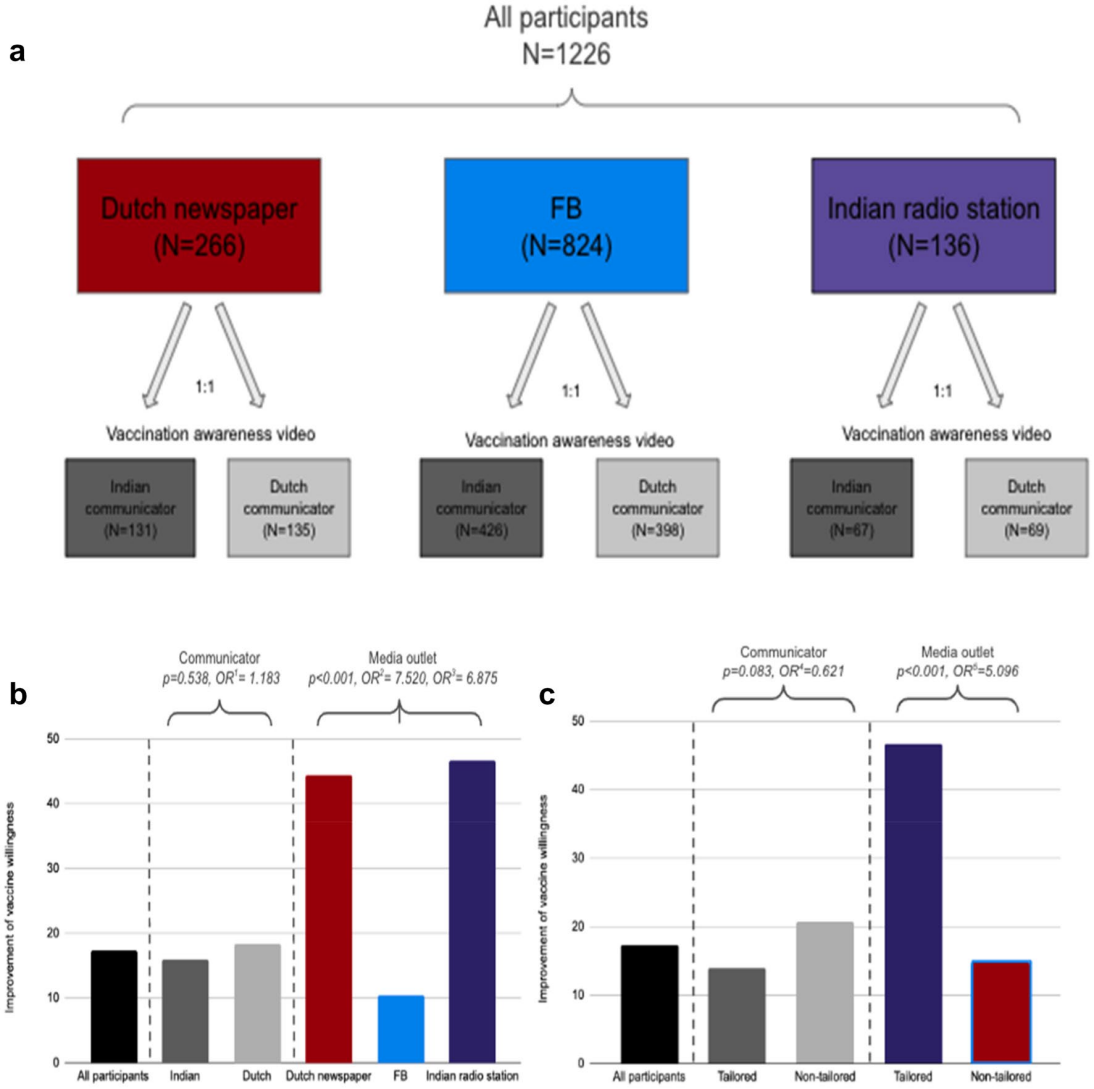
(**a)** Study flowchart. All participants were recruited from two culturally non-tailored media outlets (Dutch newspaper and Facebook (FB)) and one culturally tailored outlet (Indian radio station). (**b**) Improvement of vaccine willingness in all vaccine hesitant participants, divided by vaccine hesitant participants who watched the campaign video with an Indian versus Dutch communicator (OR^1^ = 1.183), and divided by vaccine hesitant participants who were recruited from different media outlets (Indian radio station vs. FB (OR^2^ = 7.520) and the Dutch newspaper vs. FB (OR^3^ = 6.875). (**c**) Improvement of vaccine willingness in all vaccine hesitant participants, vaccine hesitant participants who watched the campaign video with a tailored versus non-tailored communicator (OR^4^ = 0.621), and vaccine hesitant participants who have been recruited from a tailored versus non-tailored media outlet (OR^5^ = 5.096). *Note* OR^1–5^ are unadjusted Odds ratios and were generated using binary logistic regressions.

We randomized a video message (1:1) containing information highlighting the benefit of Influenza vaccines, delivered by a communicator (study physician) with either an Indian or Dutch background. The vaccination awareness videos were distributed within three different media outlets, including two culturally non-tailored (generic) media outlets and one media outlet culturally tailored to the community of Indian ancestry (mostly migrated from Suriname) in the Netherlands (Fig. 1a). The culturally tailored media outlet consisted of a local radio station called Ujala, which presents news, views and music tailored to and presented by people from Indian ancestry. The culturally non-tailored media outlets consisted of a Dutch newspaper called Algemeen Dagblad (AD) and Facebook (FB). The success of the intervention, improvement of vaccine willingness, was defined as the proportion of participants who changed from being against to pro-influenza vaccination. Within the study we compared the impact of cultural tailoring on the improvement of vaccine willingness, a. at the level of the video intervention delivered by the Indian or Dutch communicator, respectively, b. at the level of the 3 different media outlets.

A total of 1226 participants were included and recruited from the Dutch newspaper (n = 266, 9.5% migrant and 90.5% non-migrant), FB (n = 824, 11.5% migrant and 88.5% non-migrant), and the Indian radio station (n = 136, 97.1% migrant and 2.9% non-migrant). In total, 624 participants were exposed to the campaign video with the Indian communicator and 602 participants with the Dutch communicator. Demographic characteristics of the participants are summarized in Table 1.

**Table 1:** Demographic characteristics of participants at baseline, divided by Indian and Dutch communicators in our campaign video and by different media outlets.

Characteristics	All participant (N = 1226)		Communicator					Media outlet						
			Indian (N = 624)		Dutch (N = 602)		*p*-value	Dutch newspaper (N = 266)		FB (N = 824)		Indian radio station (N = 136)		*p*-value
	Median	Quartiles 1, 3 [range]	Median	Quartiles 1, 3 [range]	Median	Quartiles 1, 3 [range]		Median	Quartiles 1, 3 [range]	Median	Quartiles 1, 3 [range]	Median	Quartiles 1, 3 [range]	
Age	63.00	56.00, 69.00 [19–90]	63.00	55.00, 69.00 [19–84]	63.00	56.00, 69.00 [23–90]	0.352	62.00	54.00, 69.00 [19–90]	64.00	57.00, 70.00 [21–89]	55.50	48.00, 62.00 [19–80]	< 0.001

	Frequency	Valid (%)	Frequency	Valid (%)	Frequency	Valid (%)	*p*-value	Frequency	Valid (%)	Frequency	Valid (%)	Frequency	Valid (%)	
Gender														
Male	391	32.0	196	31.5	195	32.6	0.804	112	42.1	223	27.2	56	41.2	< 0.001
Female	827	67.7	424	68.2	403	67.3		154	57.9	594	72.5	79	58.1	
Other	3	0.2	2	0.3	1	0.2		0	0.0	2	0.2	1	0.7	
Migration background														
Migrant	251	20.6	131	21.2	120	20.1	0.626	25	9.5	94	11.5	132	97.1	< 0.001
Non-migrant	965	79.4	487	78.8	478	79.9		239	90.5	722	88.5	4	2.9	
Education														
Elementary school	75	6.1	36	5.8	39	6.5	0.562	7	2.6	60	7.3	8	5.9	< 0.001
High school														
*Prevocational secondary education*	227	18.5	118	18.9	109	18.1		39	14.7	162	19.7	26	19.1	
*Senior general secondary education*	129	10.5	62	9.9	67	11.1		25	9.4	89	10.8	15	11.0	
*Preuniversity education*	41	3.3	23	3.7	18	3.0		9	3.4	29	3.5	3	2.2	
Secondary vocational education	274	22.3	145	23.2	129	21.4		51	19.2	194	23.5	29	21.3	
Higher professional education	346	28.2	166	26.6	180	29.9		89	33.5	226	27.4	31	22.8	
University education (bachelor / master)	125	10.2	67	10.7	58	9.6		44	16.5	60	7.3	21	15.4	
Ph.D.	9	0.7	7	1.1	2	0.3		2	0.8	4	0.5	3	2.2	
Chronic disease														
Yes	770	64.5	395	65.0	375	64.1	0.755	192	72.2	523	63.5	55	53.4	0.002
No	423	35.5	213	35.0	210	35.9		74	27.8	301	36.5	48	46.6	
Do you usually get a flu shot every year?														
Yes	569	46.4	299	47.9	270	44.9	0.282	154	57.9	352	42.7	63	46.3	< 0.001
No	657	53.6	325	52.1	332	55.1		112	42.1	472	57.3	73	53.7	
Campaign video														
Indian communicator	624	50.9						131	49.2	426	51.7	67	49.3	0.724
Dutch communicator	602	49.1						135	50.8	398	48.3	69	50.7	

### At baseline

At baseline (i.e. before introducing participants to the campaign video) a total of 382 participants indicated to be vaccine hesitant (31.2% of in total 1226 participants). The highest vaccine hesitancy was in those recruited through FB (37.3%), compared to the Indian radio station (22.2%) and the Dutch newspaper (16.9%). After showing the campaign video, vaccine hesitancy decreased across the formats but remained highest in participants from FB (36.5%), followed by the Indian radio station (17.8%) and the Dutch newspaper (13.2%) (Supplementary Table 1). The overall improvement of vaccine willingness after showing the campaign video, defined as the proportion of participants having switched from against to pro-influenza vaccination was 17.3%, i.e. 66 out of 382 participants (Fig. 1b,c and Supplementary Table 2). From the 66 participants who converted to pro-influenza vaccination, 78.8% had earlier indicated in the survey to not get the flu shot usually yearly.

### At the level of communicators

In a general comparison between the two communicators (i.e. Indian and Dutch communicator) no statistically significant difference was observed with respect to the improvement of vaccine willingness in the vaccine hesitant group (18.4% vs. 16.0%, respectively, *p* = 0.538, unadjusted OR = 1.183, 95% CI 0.693–2.017, Fig. 1b and Supplementary Tables 2, 3), neither when controlling for covariates using a binary logistic regression (adjusted OR = 0.912, 95% CI 0.495–1.679, Supplementary Table 3).

When comparing cultural tailoring of the communicator (i.e. matching the background of the physician with the background of the participant) with non-tailoring of the communicators (unmatched backgrounds of the physician and the participant) we found no difference of the effect of the campaign video (13.9% for the tailored communicator vs. 20.7% for the non-tailored communicator, respectively, *p* = 0.083) (Fig. 1c and Supplementary Table 4).

These findings suggest that the participants did not show any preference for cultural tailoring of health care providers, as also was apparent from the results of our survey questions on the preference of having a culturally tailored healthcare provider (answers were tending towards ‘strongly disagree’ and ‘disagree’, Supplementary Table 2). We did observe a small difference in preference between the media outlets (the Dutch newspaper, FB and the Indian radio station) for instance in the statement ‘I value having a health care provider of a similar ethnicity/origin to myself’, with scores ranging from 1.86, 1.94 and 2.36, respectively, *p* = 0.001 (Supplementary Table 2, but the extent of the difference was small (mean scores belong to ‘disagree’). The predominantly migrant listeners to the Indian radio station tended to disagree with the importance of having a culturally tailored health care provider. These data support the findings that tailoring of the communicator might not be of decisive importance in convincing ethnic groups in our campaign.

### At the level of media outlets

In a general comparison between the three different media outlets, improvement of vaccine willingness in the vaccine hesitant group was highest in participants exposed to the campaign through the Indian radio station and the Dutch newspaper compared to campaign exposure via FB (46.7%, 44.4% and 10.4%, respectively, *p* < 0.001, unadjusted OR = 7.520 and unadjusted OR = 6.875, Fig. 1b and Supplementary Tables 2, 3). Upon controlling for different covariates (Supplementary Table 3), only media outlet was statistically significantly associated with improvement of vaccine willingness. More specifically, vaccine hesitant participants exposed to the campaign through the Indian radio station were most likely to be convinced for accepting vaccination against influenza (adjusted OR = 6.490, 95% CI 1.808–23.291), followed by the Dutch newspaper (adjusted OR = 6.103, 95% CI 2.935–12.699) compared to vaccine hesitant participants exposed to the campaign through FB. This high improvement of vaccine willingness initiated from the Indian radio station corresponds with the positive sentiment reported by the participants during the campaign in this group. A sentiment analysis of the invited feedback (comments) during the campaign (Supplementary Table 5) showed a more positive response from the Indian radio station compared with the Dutch newspaper or FB (percentage positive feedback 25.0%, 5.5% and 2.0%, *p* < 0.001, respectively). Examples of comments for each sentiment from all participants are described in Supplementary Table 6.

When comparing the effect of a culturally tailored media outlet (a media outlet with a specific audience) to culturally non-tailored media outlets (media outlets without a specific audience i.e. generic), the data show that vaccine hesitant participants exposed to the campaign delivered by a tailored media outlet (n = 30) resulted in a higher improvement of vaccine willingness compared to vaccine hesitant participants exposed to the campaign delivered by non-tailored media outlets (n = 348), 46.7% versus 14.7%, respectively, *p* < 0.001, unadjusted OR = 5.096) (Fig. 1c and Supplementary Table 4).

## Discussion

Within the scope of strategies to overcome vaccine hesitancy, our study has shown that a culturally attuned messaging strategy using a tailored media outlet for the community with Indian ancestry resulted in the highest improvement of vaccine willingness. Although the video message was similar within all three media outlets, the success of the campaign within the Indian radio station may be explained by a messaging tactic which has also been described in Foxworth et al.^15^, by emphasizing the importance of vaccination to protect the community. The positive sentiments reported by the participants who were recruited from the Indian radio station, reflect the fact that the campaign was well received, as seen by the improvement of vaccine willingness.

In contrast, our data also suggest that a generic approach in a culturally diverse population may even backfire, and harm the effect of national vaccination campaigns, given the negative sentiments reported by the participants during the campaign and the lower improvement of vaccine willingness, within the Dutch newspaper and FB groups.

To expand upon the findings of Alsan et al.^14^, in which race or ethnic messengers (physicians) aligned to the audience helped improve information-seeking behaviour specifically in Black participants, within the context of our study, it seems that cultural tailoring of the communicator/health care worker might not necessarily improve vaccine willingness.

The overall effect of the present vaccination campaign study may seem small, given the 17.3% change from being vaccine hesitant to vaccine willing. However, all participants, including those from the vaccine hesitant group were exposed only once to the intervention video. Repetitive exposure to an intervention, in which information about vaccination is provided, may help to increase the change from being hesitant to acceptance substantially^19^.

Our tailored media outlet strategy may be part of the burgeoning, so-called precision public health strategy. Precision public health is described as ‘the right intervention to the right population at the right time’^20^. The successful vaccination campaign in Alaskan Indians and American Natives^15^, and our study in the Netherlands, may serve as examples to help protect vulnerable groups.

Our study has various limitations. First, our study was conducted in the Netherlands, and thus, more evidence from across other countries is needed to substantiate the findings. Secondly, the tailored media outlet consisted of migrants recruited from only one ethnic group in the Netherlands. Although the community with Indian ancestry in the Netherlands is a large ethnic group, future research should demonstrate whether this strategy is applicable to other ethnic minorities and across other media outlets.

In conclusion, our influenza vaccination awareness campaign study resulted in improved vaccine acceptance, especially in an ethnic group recruited from a tailored media outlet, as compared to generic media outlets, among the culturally diverse population of the Netherlands. Cultural tailoring of the communicator per se did not result in enhanced vaccine willingness. Our study also suggests that a sentiment analysis may be a good predictor of the campaign’s success. Therefore, one could envision that public health campaign strategists could use the sentiment analysis within pilot interventions to shape the final version of the public health campaign. If a pilot intervention (public health message) generates predominantly negative sentiments, one could argue that the public health campaign should be adapted such that it results in positive sentiments, to have a higher chance to obtain a successful campaign outcome.

## Methods

### Data collection

The data from this study were collected between November 1st and December 31st in 2021. We used the online platform Qualtrics for the distribution of the surveys within the three different media outlets. The surveys were filled out by participants who were recruited from three different media outlets, a Dutch newspaper (called AD), Facebook (FB) and a local Indian radio station (Ujala). The total number of potential participants who clicked on the survey link, read the information sheet, and were age eligible (18 years or older) comprised 4577 participants, including 2692 participants from the Dutch newspaper, 1695 participants from FB, and 190 participants from the local Indian radio station. Participants were asked to sign an informed consent form, before participating in our campaign study. If participants did not provide informed consent, they were excluded from participation. Participants were also excluded from this study if aged < 18 years or if they did not complete the survey. In total, 1226 participants met our study criteria and were included in the campaign study. No compensation or incentives were provided to promote research participation. This study was reviewed by the Medical Ethical Review Board (METC) of the Amsterdam University Medical Center (UMC), the Netherlands, and was exempted from the need for an IRB approval. Furthermore, this study was performed in accordance with the Declaration of Helsinki, followed the CONSORT 2010 guidelines for reporting randomized trials, and was registered at ISRCTN under reference number ISRCTN13441058.

### Recruitment of participants

Participants were recruited from three different media outlets.

Participants from the Dutch newspaper (called AD) were informed about our campaign study through a news article in the (online) newspaper, in which the importance of taking the Influenza vaccination was emphasized, especially for high risk people e.g. people with a particular migration background. People were subsequently asked to participate in an online survey, originating from Qualtrics, by clicking on the attached link.

Participants recruited from FB were shown a Facebook advertisement indicating the risk of a myocardial infarction during an influenza epidemic, followed by the question “Does vaccination help?”. People were subsequently asked to fill out a survey, referring to the link to the online survey from Qualtrics.

Participants from the local Indian radio station were recruited through the radio through an interview on the subject of vaccination, short radio spots, and an advertisement on their website and Facebook page. During the live interview, broadcasted twice, listeners were invited to fill out the survey with translation support from the radio presenter. The radio spot was broadcasted for 3 weeks (4 spots per day), and voiced by an Indian communicator (Dutch cardiologist from Surinamese origin with ancestors from India), by which influenza vaccination was promoted. In both the interview and the radio spot, the importance of vaccination in relation to protection of the community was emphasized, as supported by Foxworth et al.^15^. The advertisement on the website and Facebook page from the Indian radio station was equal to the advertisement on FB. During the interview and radio spots, potential participants were directed to the website or Facebook page, where a link to the online survey, originating from Qualtrics, was shown.

### Campaign video message

The video message was based on the results from our diagnostic survey (not shown), as distributed at the beginning of the study period starting on November 1st. Through this diagnostic survey, largely based on the ‘WHO SAGE Working Group on Vaccine Hesitancy survey tool in Guatemala’^21^, we collected data about gaps in knowledge, attitude and practices concerning vaccination in the Netherlands.

The video message was voiced by a voice-over and a communicator (study physician) with either an Indian or Dutch background.

In the first part of the video message, participants received information about the importance of getting the influenza vaccine. For instance, during the influenza season 2021–2022, impairment of the immune response was expected due to the Covid-19 containment measures in the almost past two years. Furthermore, participants received information about the increased risk of developing a myocardial infarction during an influenza epidemic. Thereafter, influenza vaccine effectiveness in preventing a myocardial infarction was emphasized.

Secondly, information on vaccine accessibility, increased risk due to underlying disease, vaccine willingness, and governmental trust was provided, specifically among ethnic minorities.

Thirdly, the vaccination myth of developing Autism Spectrum Disorder after vaccination was discussed and refuted, and hesitancy about new vaccines was discussed and refuted.

The video message ended with a short story about the origin of the vaccine, which was either about inoculation (a non-Western invention) or vaccine development by Dr. Edward Jenner (a Western invention). We hypothesized that a narrative about vaccination as a non-Western invention (inoculation) would result in better improvement of vaccine willingness in the participants with a migrant background, compared to the narrative of vaccination as a Western innovation (discovered by Dr. Edward Jenner).

Participants from the Dutch newspaper received the video message with either a Dutch communicator or an Indian communicator (digitally randomized 1:1 by Qualtrics), both ending with the inoculation part. Participants recruited from FB or the local Indian radio station also received the video message with either the Dutch communicator or the Indian communicator (digitally randomized 1:1), in which, in addition, the story about inoculation or Edward Jenner was digitally randomized 1:1 as well. In this way, a total of four different video messages were digitally randomized (1:1:1:1 by Qualtrics) and were shown to participants from FB and the Indian radio station.

Side note: In the extensive analysis in this study, the video messages with the inoculation part and the Edward Jenner part, were treated as the same video message. The reason for this decision was that no difference in improvement of vaccine willingness was observed between the two versions of the video messages (i.e. the video message ending with vaccination as a Western invention (Edward Jenner part) or the version ending with vaccination as a non-Western innovation (inoculation)) within participants from FB and the Indian radio station.

In addition, we performed a regression analysis to predict improvement of vaccine willingness, controlling for age, gender, migration background, education level, presence of chronic disease, watching the inoculation part or the Edward Jenner part, communicator (Dutch or Indian) and media outlet (FB or Indian radio station), which showed that only media outlet was significantly associated with improvement of vaccine willingness. Therefore, in the extensive analysis from this study, the inoculation part and the Edward Jenner part were considered to be comparable in impact, and were treated as the same video message. In the Supplementary information section, the subanalysis of the video message with the inoculation part and Edward Jenner part is extensively described (Supplementary Table 7 and Supplementary text 1).

### Survey development

We developed a survey to measure vaccine willingness, awareness and knowledge concerning influenza vaccination among the Dutch population, from which a large part of the survey originated from our previous research work^17^. The survey contained questions to evaluate demographic information (age, gender, migration background, education, presence or absence of any chronic disease, acceptance to annual flu shot, and influenza vaccine willingness). After the initial questions, one of the campaign videos was shown to the participants with an Indian or Dutch communicator delivering the video message, followed by additional questions concerning governmental trust^22^, vaccine hesitancy (mostly adapted from the WHO SAGE Working Group on Vaccine Hesitancy survey tool in Guatemala)^21^, racial fairness and consciousness^23^, and preferences for a culturally tailored (culturally concordant) healthcare provider.

With regard to the questions about racial fairness and consciousness^23^, the scale of the answers were changed in line with previous questions, i.e. strongly disagree to strongly agree including the option ‘I don’t know’ instead of ‘never’ to ‘very often’. For the specific purpose of this study, scales to assess the domains ‘vaccine willingness’ and ‘preferences for a culturally tailored health care provider’ were developed within the research team for this particular RCT.

### Cultural tailoring

In the present study, 2 types of cultural tailoring have been discussed. First, cultural tailoring of the communicator means matching the background of the physician with the background of the participant. Cultural non-tailoring of the communicators means unmatching backgrounds of the physician and the participant. In this study we measured the effect on improvement of vaccine willingness comparing culturally tailored communicators with culturally non-tailored communicators, i.e. the Dutch communicator with a non-migrant participant and the Indian communicator with a migrant participant compared with Dutch communicator with migrant participant and Indian communicator with a non-migrant participant.

Secondly, a culturally tailored media outlet is defined as a media outlet with a specific audience, including specific views, values and norms. On the other hand, the non-tailored media outlets are without a specific audience i.e. a generic media outlet. In this study, the tailored media outlet was represented by the Indian radio station (Ujala) and the non-tailored media outlets comprised the Dutch newspaper and FB.

### Definition of improvement of vaccine willingness

Improvement of vaccine willingness was measured as the proportion of people who switched from against to pro-influenza vaccination after watching the campaign video.

### Outcomes

Vaccine willingness was measured using a 4-point Likert scale within the survey distributed within the Dutch newspaper and Facebook group, and a dichotomous scale was used within the survey distributed within the Indian radio station group. The 4-point Likert scale answers were recoded into a dichotomous scale to match with the scale used in the survey distributed within the Indian radio station group, e.g. strongly disagree and disagree were recoded into disagree; agree and strongly agree were recoded into agree. Only the dichotomous scale was used in the statistical analysis. Ordinal data was collected through 3, 4 or 5-point Likert scales. These scales were subsequently converted into numerical values (e.g. 1, 2 and 3). Question 5 regarding migration background was recoded into a binary variable, as 1 for migrant and 2 for non-migrant. Question 6 regarding education level of the participant, was recoded into a binary variable, as 0 for lower education i.e. less than high school diploma, pre-vocational secondary education and secondary vocational education, and 1 for higher education for all remaining levels of education.

### Statistical analyses

Continuous data were analyzed with the Mann–Whitney U test, or with the Kruskal–Wallis-test in case of comparing continuous data within three groups (three different media outlets). Binary, ordinal or nominal data were analyzed with the Chi-Square test. The outcome measure ‘improvement of vaccine willingness’ was defined as participants who answered ‘disagree’ to the question whether they were pro-influenza vaccination before watching the campaign video, and switched to ‘agree’ after watching the campaign video. Improvement of vaccine willingness, which means converted from against to pro-influenza vaccination, was coded by 1 and no improvement was coded by 0. Differences in improvement of vaccine willingness between participants who watched our campaign video with a Dutch versus Indian communicator or between different media outlets were analyzed with the Chi-Square test. A binary logistic regression was performed to analyze unadjusted and adjusted (with different predictors in the model) odds ratios concerning improvement of vaccine willingness. Within the adjusted regression model to predict improvement of vaccine willingness in the vaccine hesitant group, we controlled for media outlet, communicator, age, gender, migration background, education level, and chronic disease. We did not control for ‘flu shot each year’, because this relationship with vaccine willingness was too obvious. Overall, results are reported as frequencies (with percentages, and minimum and maximum) or medians (with quartiles 1 and 3, and minimum and maximum).

#### Sentiment analysis

We performed a sentiment analysis, where we extracted all comments from the feedback section from our survey. In order to classify comments as either negative, neutral or positive, three researchers individually assigned comments to one of the three different sentiments based on words used by participants, and according to specific words indicated in the work of Pudaruth et al.^24^. The sentiment of a comment was subjectively scored as either negative, neutral or positive, as a reaction to the survey or the subject of vaccination in general. If discrepancy occurred between the sentiment score from each individual researcher to a specific comment, the sentiment was assigned to neutral. Sentiments were coded as 0 for negative, 1 for neutral and 2 for positive, and were analyzed with the Chi-Square test.

## Supplementary Information

Supplementary Information.

## Data Availability

The data that support the findings of this study are available from the corresponding author upon request.

## References

[1] Ferdinands JM (2021). Does influenza vaccination attenuate the severity of breakthrough infections? A narrative review and recommendations for further research. Vaccine.

[2] Barnes M (2015). Acute myocardial infarction and influenza: A meta-analysis of case-control studies. Heart.

[3] Habib GL, Yousuf H, Narula J, Hofstra L (2021). Call to action: Cardiologists should promote influenza vaccination. Neth. Heart J..

[4] Heins, M., Korevaar, J., Knottnerus, B. & Hooiveld, M. Monitor vaccinatiegraad nationaal programma grieppreventie (NPG) 2021. *Nivel.* 3–41 (2022).

[5] No author reported. Influenza vaccination coverage and effectiveness. WHO. https://www.who.int/europe/news-room/fact-sheets/item/influenza-vaccination-coverage-and-effectiveness​​ (2018).

[6] Flu Vaccination Coverage, United States, 2019–20 Influenza Season Centers for Disease Control and Prevention, National Center for Immunization and Respiratory Diseases (NCIRD), updated 03/03/2022. Available from: cdc.gov/flu/fluvaxview/coverage-1920estimates.htm#additional (2020).

[7] Quinn SC, Andrasik MP (2021). Addressing vaccine hesitancy in BIPOC communities—Toward trustworthiness, partnership, and reciprocity. N. Engl. J. Med..

[8] Stronks, K. *et al.* Bevolkingsgroepen met migratieachtergrond zwaarder getroffen door covid-19 https://www.zonmw.nl/fileadmin/zonmw/documenten/Corona/Policy_brief_Etniciteit_en_COVID-19_mei_2021.pdf (2021).

[9] Yousuf H (2021). Dutch perspectives toward governmental trust, vaccination, myths, and knowledge about vaccines and COVID-19. JAMA Netw. Open..

[10] Heins, M., Hooiveld, M. & Korevaar, J. Monitor vaccinatiegraad nationaal programma grieppreventie 2019. *Nivel*. 3–36 (2020).

[11] Heins, M., Hooiveld, M. & Korevaar, J. Monitor vaccinatiegraad nationaal programma grieppreventie 2020. *Nivel*. 3–36 (2021).

[12] Webb Hooper M, Napoles AM, Perez-Stable EJ (2020). COVID-19 and racial/ethnic disparities. JAMA.

[13] Alsan M, Stantcheva S, Yang D, Cutler D (2020). Disparities in Coronavirus 2019 reported incidence, knowledge, and behavior among US adults. JAMA Netw. Open..

[14] Alsan M (2021). Comparison of knowledge and information-seeking behavior after general COVID-19 public health messages and messages tailored for black and latinx communities : A randomized controlled trial. Ann. Intern. Med..

[15] Foxworth R (2021). Covid-19 vaccination in American Indians and Alaska Natives—Lessons from effective community responses. N. Engl. J. Med..

[16] Yousuf H (2020). Association of a public health campaign about Coronavirus disease 2019 promoted by news media and a social influencer with self-reported personal hygiene and physical distancing in the Netherlands. JAMA Netw. Open..

[17] Yousuf H (2021). A media intervention applying debunking versus non-debunking content to combat vaccine misinformation in elderly in the Netherlands: A digital randomised trial. EClinicalMedicine.

[18] van der Linden S (2022). Misinformation: Susceptibility, spread, and interventions to immunize the public. Nat. Med..

[19] d'Alessandro E (2012). Determinants of refusal of A/H1N1 pandemic vaccination in a high risk population: A qualitative approach. PLoS ONE.

[20] Arnold C (2022). Spurred by covid, public health gets precise. Nature.

[21] Domek GJ (2018). Measuring vaccine hesitancy: Field testing the WHO SAGE working group on vaccine hesitancy survey tool in guatemala. Vaccine.

[22] Freimuth VS, Musa D, Hilyard K, Quinn SC, Kim K (2014). Trust during the early stages of the 2009 H1N1 pandemic. J. Health Commun..

[23] Quinn SC (2017). Exploring racial influences on flu vaccine attitudes and behavior: Results of a national survey of White and African American adults. Vaccine.

[24] Pudaruth S, Moheeputh S, Permessur N, Chamroo A (2018). Sentiment analysis from facebook comments using automatic coding in NVivo 11. ADCAIJ.

