# Supplementary material for: The importance of cultural tailoring of communicators and media outlets in an influenza vaccination awareness campaign: a digital randomized trial
Source: Sci Rep. 2023 Feb 16;13:1744. doi: 10.1038/s41598-023-27910-y (PMC9935604; doi:10.1038/s41598-023-27910-y)
Supplement: Supplementary file 1 — Supplementary Information. [file 41598_2023_27910_MOESM1_ESM.docx]

**Supplementary information**

**The importance of cultural tailoring of communicators and media outlets in an Influenza vaccination awareness campaign: A digital randomized trial**

G.L. Habib^a^, H. Yousuf^a^, L. Bredius^a^, N. Bindraban^a^, M. Winter^a^, E. Scherder^b^, S. van der Linden^c^, J. Narula^d^, L. Hofstra*^a^

^a^Department of Cardiology, Amsterdam UMC, Amsterdam, The Netherlands

^b^Department of Clinical Neuropsychology, VU, Amsterdam, the Netherlands. ^c^Department of Psychology, School of Biology, University of Cambridge, Cambridge, United Kingdom.

^d^Mount Sinai Heart, Icahn School of Medicine at Mount Sinai, New York, New York.

*Corresponding author: Leonard Hofstra

l.hofstra@cardiologiecentra.nl

+31(0)6-44295945

|  | **Respondents**  **frequencies** | **Influenza Vaccine Hesitancy**  **Pre-intervention**  **frequencies (%)** | **Influenza Vaccine**  **Hesitancy**  **Post-intervention**  **frequencies (%)** |
| --- | --- | --- | --- |
| **Total** | 1225 | 382 (31.2) | 360 (29.4) |
| Indian communicator | 624 | 181 (29.0) | 173 (27.7) |
| Dutch communicator | 601 | 201 (33.4) | 187 (31.1) |
| **AD** |  |  |  |
| Total | 266 | 45 (16.9) | 35 (13.2) |
| **FB** |  |  |  |
| Total | 824 | 307 (37.3) | 301 (36.5) |
| **Indian radio station** |  |  |  |
| Total | 135 | 30 (22.2) | 24 (17.8) |

Supplementary table 1: Influenza vaccine hesitancy pre- and post-intervention among all participants and divided by different communicators and media outlets.

|  | **All participants** | | | **Communicator** | | | | **P-value** | **Media outlet** | | | | | | | | | **P-value** |
| --- | --- | --- | --- | --- | --- | --- | --- | --- | --- | --- | --- | --- | --- | --- | --- | --- | --- | --- |
|  | (N=1226) | | | **Indian**  (N=624) | | **Dutch**  (N=602) | |  | **Dutch newspaper**  (N=266) | | | **FB**  (N=824) | | | **Indian radio station**  (N=136) | | |  |
|  | **Respondents** | **%** | | **Respondents** | **%** | **Respondents** | **%** |  | **Respondents** | **%** | | **Respondents** | **%** | | **Respondents** | **%** | |  |
| **Improvement of vaccine willingness** | 66 (out of 382) | 17.3% | | 29 (out of 181) | 16.0% | 37 (out of 201) | 18.4% | 0.538 | 20 (out of 45) | 44.4% | | 32 (out of 307) | 10.4% | | 14 (out of 30) | 46.7% | | <0.001 |
| **Questions** |  | | | | | | | | | | | | | | | | | |
| **Racial / ethnic tailoring** |  | **mean** | **SD** |  | | | | |  | **mean** | **SD** |  | **mean** | **SD** |  | **mean** | **SD** |  |
| 'I value having a health care provider of a similar ethnicity/origin to myself' |  | 1.97 | 1.077 |  |  |  |  |  |  | 1.86 | 1.009 |  | 1.94 | 1.055 |  | 2.36 | 1.245 | 0.001 |
| 'I think a health care provider of a similar ethnicity/origin as me can provide better care' |  | 1.82 | 0.942 |  |  |  |  |  |  | 1.64 | 0.784 |  | 1.80 | 0.918 |  | 2.28 | 1.197 | <0.001 |
| 'If my healthcare provider is of a similar ethnicity/origin as I am, I am more satisfied with the communication during an appointment' |  | 2.24 | 1.164 |  |  |  |  |  |  | 2.24 | 1.192 |  | 2.22 | 1.146 |  | 2.34 | 1.219 | 0.308 |
| ‘If my healthcare provider is of a similar ethnicity/origin as I am, I get better information about the treatment, disease, etc.’ |  | 2.00 | 1.004 |  |  |  |  |  |  | 1.81 | 0.861 |  | 2.01 | 0.999 |  | 2.30 | 1.207 | <0.001 |
| 'The Dutch government acts in the best interest of people of my ethnicity / origin' |  | 2.85 | 1.190 |  |  |  |  |  |  | 3.25 | 1.280 |  | 2.72 | 1.142 |  | 2.84 | 1.124 | <0.001 |
| **‘**Racism makes a difference in getting access to certain medicines or treatments in certain ethnic groups’ |  | 2.19 | 1.103 |  |  |  |  |  |  | 2.04 | 1.096 |  | 2.19 | 1.084 |  | 2.51 | 1.174 | <0.001 |

Supplementary table 2: Improvement of vaccine willingness in the vaccine hesitant group and perspectives on cultural tailoring of health care providers in all participants and divided by different media outlets.

| **Improvement of vaccine willingness** | **OR** | **CI** |
| --- | --- | --- |
| **Unadjusted** | | |
| Communicator (Dutch vs. Indian) | 1.183 | 0.693 - 2.017 |
| **Unadjusted** | | |
| Media outlet |  |  |
| *Indian radio station vs. FB* | 7.520 | 3.361 - 16.824 |
| *Dutch newspaper vs. FB* | 6.875 | 3.439 - 13.743 |
| *Indian radio station vs. Dutch newspaper* | 1.094 | 0.433 - 2.765 |
| **Adjusted** | | |
| Media outlet |  |  |
| *Indian radio station vs. FB* | 6.490 | 1.808 - 23.291 |
| *Dutch newspaper vs. FB* | 6.103 | 2.935 - 12.688 |
| *Indian radio station vs. Dutch newspaper* | 1.063 | 0.257 - 4.396 |
| Communicator (Dutch vs. Indian) | 0.912 | 0.495 - 1.679 |
| Age | 1.001 | 0.971 - 1.031 |
| Gender (female vs. male) | 1.579 | 0.787 - 3.171 |
| Migration background (non-migrant vs. migrant) | 0.482 | 0.196 - 1.187 |
| Education (high vs. low) | 1.033 | 0.563 - 1.896 |
| Chronic disease (yes vs. no) | 1.777 | 0.930 - 3.394 |

Supplementary table 3: Binary logistic regression of improvement of vaccine willingness in the vaccine hesitant group (unadjusted) and controlled for different covariates (adjusted).

|  | **Communicator** | | | | | | | **Media outlet** | | | | |  | | |
| --- | --- | --- | --- | --- | --- | --- | --- | --- | --- | --- | --- | --- | --- | --- | --- |
|  | Tailored  (n=194) | | Non-tailored  (n=184) | |  | | | Tailored  (n=30) | | Non-tailored (n=348) | |  | | |  |
|  | frequencies | % | frequencies | % | p-value | OR (unadjusted)* | CI* | frequencies | % | frequencies | % | p-value | OR (unadjusted)* | CI* |  |
| **Improvement of vaccine willingness** | 27 | 13.9 | 38 | 20.7 | 0.083 | 0.621 | 0.362 - 1.067 | 14 | 46.7% | 51 | 14.7% | <0.001 | 5.096 | 2.344 - 11.076 |  |

Supplementary table 4: Cultural tailoring versus non-tailoring of communicators and media outlets concerning improvement of vaccine willingness in the vaccine hesitant group.

*values (unadjusted) were assessed using binary logistic regressions.

|  | **Media outlet** | | | | | | |
| --- | --- | --- | --- | --- | --- | --- | --- |
| **Sentiment** | Dutch newspaper | | FB | | Indian radio station | | P-value |
|  | frequency | % | frequency | % | frequency | % |  |
| Negative | 20 | 40.8 | 79 | 43.6 | 4 | 14.3 |  |
| Neutral | 28 | 57.1 | 92 | 50.8 | 17 | 60.7 |  |
| Positive | 1 | 2.0 | 10 | 5.5 | 7 | 25.0 |  |
| Total reactions | 49 |  | 181 |  | 28 |  | <0.001 |

Supplementary table 5: Sentiment analysis of 3 different media outlets, among all participants who gave feedback during the campaign.

| **Sentiment analysis of comments** |
| --- |
| **Negative comments** |
| You can't say anywhere why you are against the indirect forced poison injection. |
| Got two COVID vaccinations and definitely not going to get the 3rd. |
| Bull shit. This is only a benefit for the pharmaceutical mafia and the doctors paid by this mafia. |
| Tribunals will come. |
| The government's compulsion to vaccinate must be stopped. We live in a dictatorship. |
| **Neutral comments** |
| Question not completely clear. |
| I already knew all the facts mentioned in the video. |
| What does ethnicity have to do with the heart? |
| Why can you still be infected after the booster? |
| The government should provide more open and transparent information about vaccines in general. |
| **Positive comments** |
| Instructive questionnaire! |
| Good initiative, hopefully this research will help to give the vaccine willingness a "boost". |
| Good luck and thanks for the information. |
| I still need to get vaccinated against the flu and get it done soon, thank you. |
| Hopefully more people will get vaccinated, so important. |

Supplementary table (6): Examples of comments for each sentiment (negative, neutral or positive) originating from the invited feedback during our campaign from all participants.

| **Improvement of vaccine willingness** | **P-value** | **OR** | **CI** |
| --- | --- | --- | --- |
| **Unadjusted** | | | |
| Video message (Inoculation vs. Edward Jenner) | 0.080 | 1.787 | 0.934 – 3.419 |
| **Adjusted** | | | |
| Video message (Inoculation vs. Edward Jenner) | 0.466 | 1.307 | 0.636 – 2.686 |
| Media outlet |  |  |  |
| *Indian radio station vs. FB* | 0.005 | 6.805 | 1.780 – 26.019 |
| Communicator (Dutch vs. Indian) | 0.574 | 1.223 | 0.607 – 2.463 |
| Age | 0.873 | 1.003 | 0.968 – 1.039 |
| Gender (female vs. male) | 0.581 | 1.244 | 0.573 – 2.700 |
| Migration background (non-migrant vs. migrant) | 0.197 | 0.523 | 0.196 – 1.399 |
| Education (high vs. low) | 0.999 | 0.999 | 0.493 – 2.026 |
| Chronic disease (yes vs. no) | 0.073 | 1.981 | 0.938 – 4.184 |

Supplementary table 7: Subanalysis of the video message with the inoculation part and the Edward Jenner part using binary logistic regressions to predict improvement of vaccine willingness in the vaccine hesitant group within FB and the Indian radio station (unadjusted) and controlled for different covariates (adjusted).

**Supplementary text 1: subanalysis video message with inoculation part and Edward Jenner part.**

In total, 960 participants were recruited from FB and the Indian radio station, including 337 participants (35.1%) who were vaccine hesitant. After exposure to any of the video messages (four different videos), 46 vaccine hesitant participants (13.6%) showed improvement of vaccine willingness.

Within FB and the Indian radio station, 496 out of 960 participants (51.7%) watched the video message including the inoculation part (consisting of 424 participants (85.5%) from FB and 72 participants (14.5%) from the Indian radio station). A total of 464 out of 960 participants (48.3%) watched the Edward Jenner part (consisting of 400 participants (86.2%) from FB and 64 participants (13.8%) from the Indian radio station).

Improvement of vaccine willingness in participants who watched the inoculation part was not significantly different from participants who watched the Edward Jenner part, 16.8% (30 out of 179) vs. 10.1% (16 out of 158), respectively, p=0.077.

A regression analysis, adjusting for age, gender, migration background, education level, presence of chronic disease, watching the inoculation part or the Edward Jenner part, communicator (Dutch or Indian) and media outlet (FB or Indian radio station), showed that only media outlet was significantly associated with improvement of vaccine willingness. More specifically, vaccine hesitant participants exposed to the campaign through the Indian radio station were more likely to be convinced to accept vaccination against influenza compared to vaccine hesitant participants from FB (adjusted OR 6.805, 95% CI 1.780 - 26.019).

In conclusion, improvement of vaccine willingness in participants from FB and the Indian radio station was not significantly different when comparing the video message with the inoculation part to the Edward Jenner part. Therefore, the impact of the video message with the inoculation part and the Edward Jenner part may be considered equal. According to the regression analysis, only media outlet was significantly associated with improvement of vaccine willingness.
